# Biomarkers associating endothelial Dysregulation in pediatric-onset systemic lupus erythematous

**DOI:** 10.1186/s12969-019-0369-7

**Published:** 2019-10-24

**Authors:** Wan-Fang Lee, Chao-Yi Wu, Huang-Yu Yang, Wen-I Lee, Li-Chen Chen, Liang-Shiou Ou, Jing-Long Huang

**Affiliations:** 1 Division of Allergy, Asthma, and Rheumatology, Department of Pediatrics, Chang Gung Memorial Hospital Linko branch, Taoyuan, Taiwan; 2 Chang Gung University, College of Medicine, Taoyuan, Taiwan; 3 Department of Nephrology, Chang Gung Memorial Hospital Linko branch, Taoyuan, Taiwan

**Keywords:** Systemic lupus erythematosus, Biomarkers, Endothelial cell

## Abstract

**Background/purpose:**

Endothelium is a key element in the regulation of vascular homeostasis and its alteration can lead to the development of vascular diseases. Systemic lupus erythematosus (SLE) is a systemic autoimmune disease with potential extensive vascular lesions, involving skin vessels, renal glomeruli, cardiovascular system, brain, lung alveoli, gastrointestinal tract vessels and more. We aimed to assess endothelial dysregulation related biomarkers in pediatric-onset SLE (pSLE) patient serum and elucidate its correlation with their clinical features, laboratory parameters, and the overall disease activity.

**Methods:**

Disease activities were evaluated by SLE disease activity index (SLEDAI). Patient characteristics were obtained by retrospective chart review. Six biomarkers associated with endothelial dysregulation, including Angiopoietin-1 (Ang-1), Angiopoietin-2 (Ang-2), Tie2, Vascular endothelial growth facto**r** (VEGF), thrombomodulin, and a disintegrin-like and metalloprotease with thrombospondin type 1 motif (ADAMTS13) were tested through enzyme-linked immunosorbent assay (ELISA) measurement.

**Results:**

This study comprised 118 pSLE patients. Data from 40 age-matched healthy controls were also obtained. The mean diagnostic age was 13 ± 4.12 years-old and 90.7% are females. Serum levels of VEGF, Tie2, thrombomodulin were significantly higher while serum ADAMTS13 was lower in active pSLE patients when compared to those with inactive diseases (all *p* < 0.05). In organ specific association, serum thrombomodulin level was higher in pSLE patient with renal involvement, and serum ADAMTS13 levels was negatively associated with neurological involvement (*p* < 0.05). A cutoff of thrombomodulin at 3333.6 pg/ml best correlated renal involvement. (AUC = 0.752, *p* < 0.01).

**Conclusion:**

Endothelial dysregulation associating proteins seems to be potent biomarkers for pSLE activity as well as organ involvement in pSLE patients. These biomarkers may be beneficial in understanding of the vascular pathogenesis and disease monitoring.

## Background

Systemic lupus erythematosus (SLE) is a prototypic autoimmune disease with potential extensive vasculitis and angiopathy [1]. Cutaneous vasculitis, glomerulonephritis, cardiopulmonary, cerebrovascular, and gastrointestinal damages are some of the most characteristic lesions of SLE vascular injury [2]. Additionally, a significant proportion of patients with SLE have evidence of subclinical vascular disease, which may be prone to atherosclerosis formation [3]. Cardiovascular disease and inflammation involving vital organs, including central nervous system (CNS) vasculitis, thrombotic microangiopathy (TMA), antiphospholipid syndrome and retinal vasculitis modulated by endothelial cell dysfunction contribute the morbidity and mortality in SLE [4–8].

Pediatric-onset SLE (pSLE) represents 10–20% of all SLE cases and is associated with more severe disease, including more-rapid damage accrual, than adult-onset SLE [9]. In 2008, LB Tucker, et al. found that patients with adolescent-onset SLE had more active disease during the entire follow-up period as measured by the revised Systemic Lupus Activity Measure (SLAM-R) and physician rating of disease activity, although these differences were not statistically significant. Moreover, patients with adolescent-onset SLE were found to have significantly higher occurrence of renal and neurological involvements at time of diagnosis when compared with adult-onset lupus patients [10]. N Ambrose et al. later pointed out that there’s an aggressive phenotype of disease in patients with childhood and adolescence onset SLE. The standardized mortality ratio was 18.3 in cSLE and 3.1 in adult-onset [11]. The majority of pSLE patients will have developed damage within 5–10 years of disease onset, most frequently involving the musculoskeletal, ocular, renal and neuropsychiatric systems [9]. Premature atherosclerosis have become increasingly prevalent morbidities in pSLE patients. Early atherosclerosis leads to a considerable rise in cardiovascular and cerebrovascular events [12].

Vasculopathy in SLE is possibly mediated by various mechanisms, including cell-mediated cytotoxicity of the endothelium; disposition of immune complexes, anti-endothelial cell antibodies, anti-double stranded DNA (dsDNA) antibodies; and the proinflammatory effect of various cytokines (eg, tumour necrosis factor (TNF-a) and anti-phospholipid antibodies [1]. Dyslipidemia; hyperhomocystenemia; and an acute stress injury of the vascular endothelium may be followed by Endothelial Cell (EC) apoptosis [13]. Endothelial dysregulation not only is an early marker of atherogenesis, the imbalance between vasodilation and vasoconstriction, as well as blood clot formation and fibrinolysis can both lead to endothelial cell damage and clinical vasculopathy [14]. Moreover, failure in smooth muscle cell proliferation, migration and damage repairing may also aggravated endothelial dysfunction [13, 14].

Endothelium is a key element in the regulation of vascular homeostasis and its alteration is a precursor of vascular disease. To elucidate the association and possible pathogenesis underlining SLE disease activity and organ involvement in a vascular aspect, we carefully examined a panel of endothelial dysregulation biomarkers in patients with pSLE. Considering Ang-1, Ang-2 and Tie2 were rather important in the homeostasis of endothelial cell activation and inflammation; VEGF was essential for endothelial cell survival; thrombomodulin as a symbol of endothelial cell injury and ADAMTS13’s role in the endothelial cells related thrombotic events, we aimed to assess these proteins in patients with pSLE and dissect its correlation with patients’ clinical features, laboratory parameters, and the overall disease activity.

## Methods

### Patients

A total of 118 pediatric-onset patients with SLE were recruited from the Department of Allergy, asthma and Rheumatology of Chang Gung Memorial Hospital (CGMH) between 2015 and 2018. It is a cross sectional study. The patients with SLE had met the 1997 American College of Rheumatology (ACR) criteria for the classification of SLE [15]. Patient characteristics were obtained by retrospective chart review. Patients with malignant diseases and acute infections were excluded. Disease activity was assessed by the SLE Disease Activity Index (SLEDAI) [16, 17]. Clinical and immunological characteristics, as well as renal histopathological composition of patients with pSLE are shown in Tables 1 and 2. Medication used at time of recruitment were also listed. A total of 40 age-matched healthy controls were also recruited. Written informed consent was collected from all the subjects participating in the study and/or their legal guardian. The research was in compliance with the Declaration of Helsinki and was approved by the CGMH Institutional Review Board (IRB No.:105-01678A3C501).

**Table 1:** Patients Characteristics, and Laboratory Data

Patient Characteristics	
No of participants, n	118
Female/Male, n (%)	107/11 (90.7)
Age at diagnosis, years	13.44±4.12
Current age, years	20.4±6.32
Laboratory data	
C3, mg/dL	67.87±30.37
C4, mg/dL	11.40±11.41
Positive ANA, n(%)	106/118 (89.8)
Anti-dsDNA, unit/mL	312.99±182.76
Medication	
Prednisolone	111/118 (94.10)
Azathioprine	58/118(50.00)
Hydroxychloroquine	50/118 (42.40)
Mycophenolic acid	27/118 (22.90)
Methylprednisolone pulses	21/118 (17.80)
Cyclophosphamide	15/118 (12.70)

**Table 2:** Organ Involvement in SLE Patients

Organ involvement, n (%)	
Neurological symptoms	15/118 (12.71)
Vasculitis	4/118 (3.40)
Arthritis	8/118 (6.80)
Myositis	2/118 (1.70)
Skin	23/118 (19.50)
Mucosa	15/118 (12.70)
Serositis	8/118 (6.80)
Immunology	93/118 (78.80)
Hematology	24/118 (20.30)
Renal; WHO lupus nephritis, n (%)	
Class I	0 /73 (0.0)
Class II	8 /73 (10.9)
Class III	12 /73 (16.4)
Class IV	44/73(60.3)
Class V	4 /73 (5.5)
Class VI	0 /73 (0.0)
Class III+V	3 /73 (4.1)
Class IV+V	2 /73 (2.7)

### Definition of disease status and organ involvement

According to the SLEDAI, patients in this cohort were subdivided into an “active” (SLEDAI> 12) and an “inactive” disease group (SLEDAI≤12). Renal pSLE group was defined having one or more of the following: protein in urine > 0.5 g/24 h, hematuria, pyuria, urinary casts (red cell, hemoglobin, granular, tubular, or mixed casts), and/or abnormal serum creatinine concentrations [17] Neurologic manifestations was defined as in the form of cognitive dysfunction, severe anxiety, psychosis, organic brain syndrome, or optic neuropathy [17].

### Protein measurement

Serum levels of Ang-1, Ang-2, Tie2, VEGF, Thrombomodulin and ADAMTS13 were measure by a commercially available ELISA kit obtained from R&D and operated according to the manufacturer’s instructions. In brief, monoclonal antibodies specific for human Ang-1, Ang-2, Tie2, VEGF, Thrombomodulin and ADAMTS13 have been pre-coated onto a microplate. Standards and samples are pipetted into the wells and any targeted protein present will be bind to the immobilized antibody. After washing away any unbound substances, an enzyme-linked monoclonal antibody specific for our target protein is added to the wells. Following a wash to remove any unbound antibody-enzyme reagent, a substrate solution is added to the wells and color develops in proportion to the amount of proteins bound in the initial step. The color development is stopped and the intensity of the color is measured.

### Statistical analysis

The softwares SPSS (v.20.0) was used for the analyses. Differences between patient groups and healthy controls were evaluated using analysis of variance (ANOVA). The significance level of *p* < 0.05 was considered statistically significant for comparisons before Bonferroni test. For multiple comparisons analysis the considered significance levels were *p* < 0.05.

Correlations between biomarkers concentrations and parameters of disease activity were calculated with the Spearman test. The Pearson correlation coefficient was calculated. All test were conducted two sided at an α level of 0.05. The receiver operating characteristic (ROC) curve analysis was used to get the best area under curve compared to conventional anti-dsDNA for diagnosing disease activity and organ involvement.

## Results

Among the 118 pediatric-onset SLE patients, 90.7% (M:F = 11: 107) are females. The mean diagnostic age was 13 ± 4.12 years-old and the average follow up period is 7 ± 2.21 years. As for organ involvement, 15 patients (12.71%) had neurological manifestations, 60 patients (50.8%) has renal involvement, and 73 (61.86%) of those with lupus nephritis underwent renal biopsy. 56 (76.71%) of them suffered class III and IV lupus nephritis. (Table 1 and 2).

### Correlation of biomarkers with pSLE disease activity

**Fig. 1:**
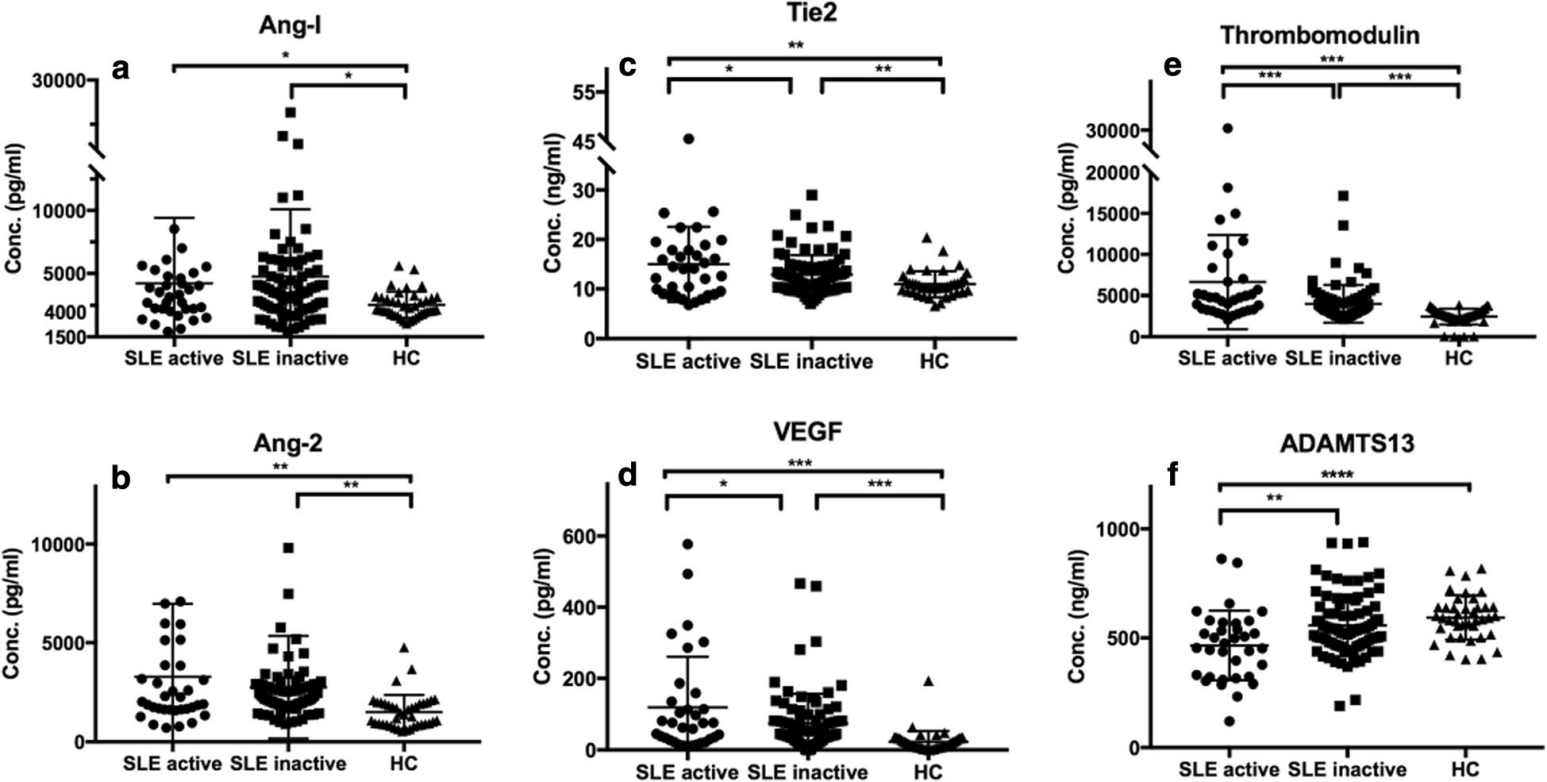
Markers Associated with Disease Activity Using Cutoff level of SLEDAI 12 (active v.s. inactive). Almost all the markers (**b**, **c**, **d**, **e**) except ADAMTS13 (**f**) and Ang-1 (**a**) are higher in pSLE patients compared to healthy controls. Serum Tie2 (**c**), VEGF(**d**), and Thrombomodulin (**e**) are correlated with high disease activity in pSLE; Whereas, serum levels of ADAMTS13 (F) is significantly lower in active pSLE patients compared to inactive pSLE. *p < 0.05, **p < 0.01, ***p < 0.001, ****p < 0.0001

**Fig. 2:**
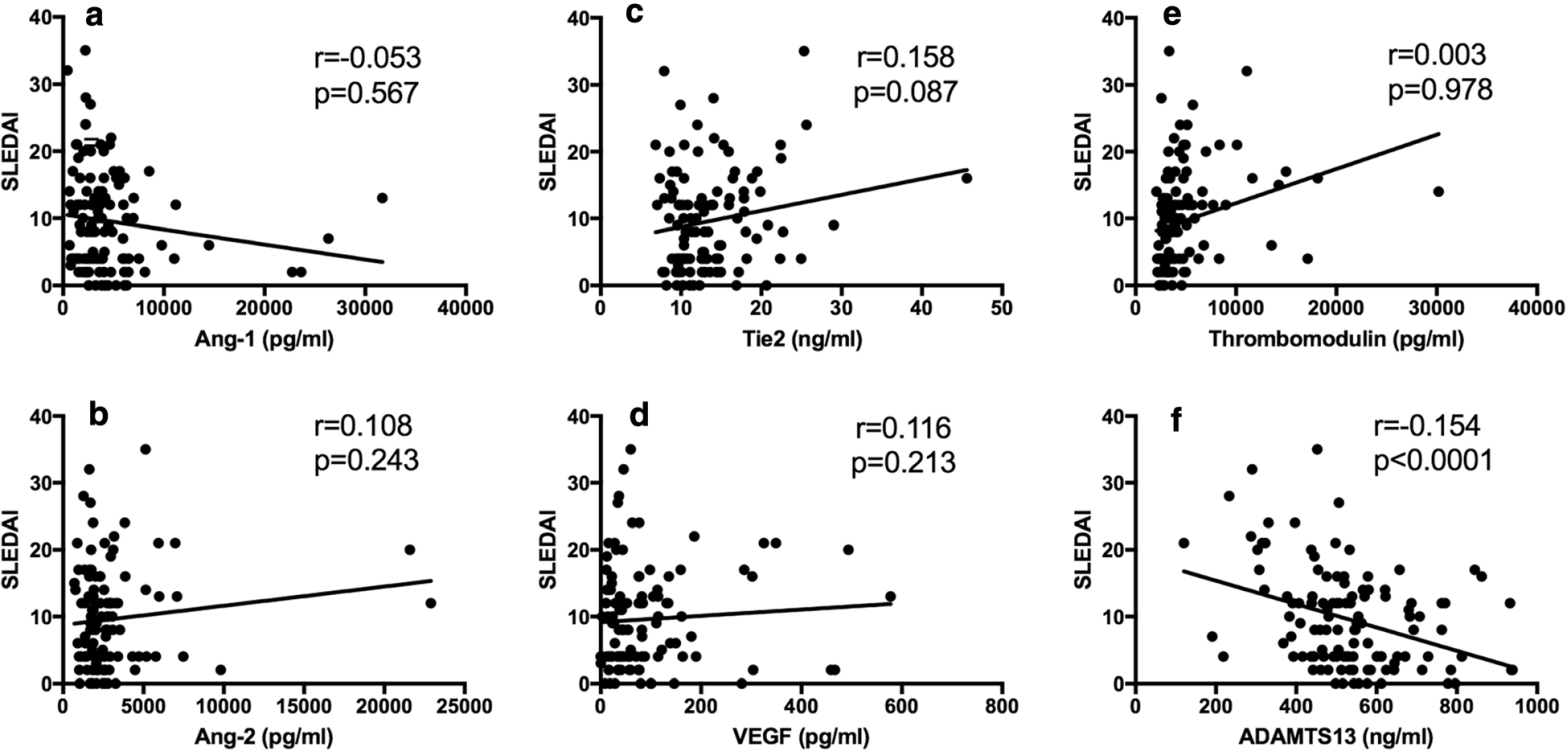
Markers Associated with Disease Activity (SLEDAI). Serum Ang-2(**b**), Tie2(**c**), VEGF(**d**), Thrombomodulin(**e**), positively correlates with SLEDAI. Ang-1(**a**) negatively correlates with SLEDAI, but not in statistically significance; whereas ADAMTS 13(**f**) has significantly negative correlation with SLEDAI

All the serum markers except ADAMTS13 are significantly higher in pSLE patients as compared to healthy controls (all *p* < 0.05). Concentrations of circulating Tie2, VEGF, and thrombomodulin are significantly increased in active pSLE patients (SLEDAI > 12) compared to inactive pSLE patients while ADAMTS13 is significantly decreased *p* = 0.049, *p* = 0.034, *p = 0.0004*, *p = 0.0025*, respectively) (Fig. 1). Further analyzed the association of the biomarkers with SLEDAI, we found that only ADAMTS 13 inversely associated with SLEDAI significantly (*p* < 0.0001) (Fig. 2). Serum Ang-2, Tie2, VEGF, and thrombomodulin are not significantly correlated with SLEDAI (*p* = 0.087, *p* = 0.243, *p* = 0.213 and *p* = 0.978, respectively). A similar trend but less significant were noted when 7 was used for cutoff when analyzed. As shown in (Additional file 1: Figure S1), the level of Tie2 and VEGF in active SLE were still higher but did not reach statistical differences when compared to the inactive pSLEs. Similar finding was also noted with ADAMTS13 at a cutoff level of 7.

**Fig. 3:**
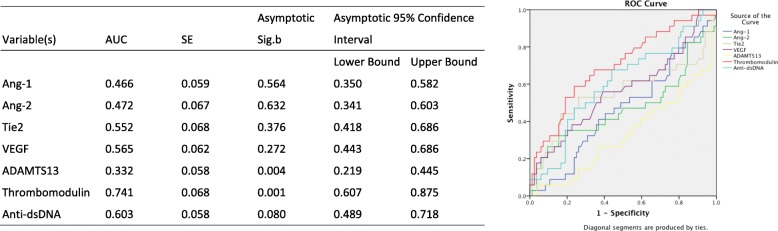
Predictive Value of Biomarkers in Disease Activity Compared to Anti-dsDNA. ROC curve analysis for each biomarkers as well as anti-dsDNA in prediction of active pSLE patients. Red: Thrombomodulin. Light blue: Anti-dsDNA

Compared to anti-dsDNA, a autoantibody classically known to well correlate pSLE disease activity [18–20], we found that thrombomodulin at a cutoff of 4346.79 pg/ml had an even better predictive value (*p* < 0.01; AUC = 0.74 vs 0.60). None of the other markers had a predictive value (all AUC < 0.57) (Fig. 3).

Considering that SLE vasculopathy in different organs may be resulted from distinct pathogenesis with diverse mechanism of endothelial dysregulation, the use of variable biomarkers in organ involves were analyzed.

### Biomarkers associated with neurological involvement in pSLE

**Fig. 4:**
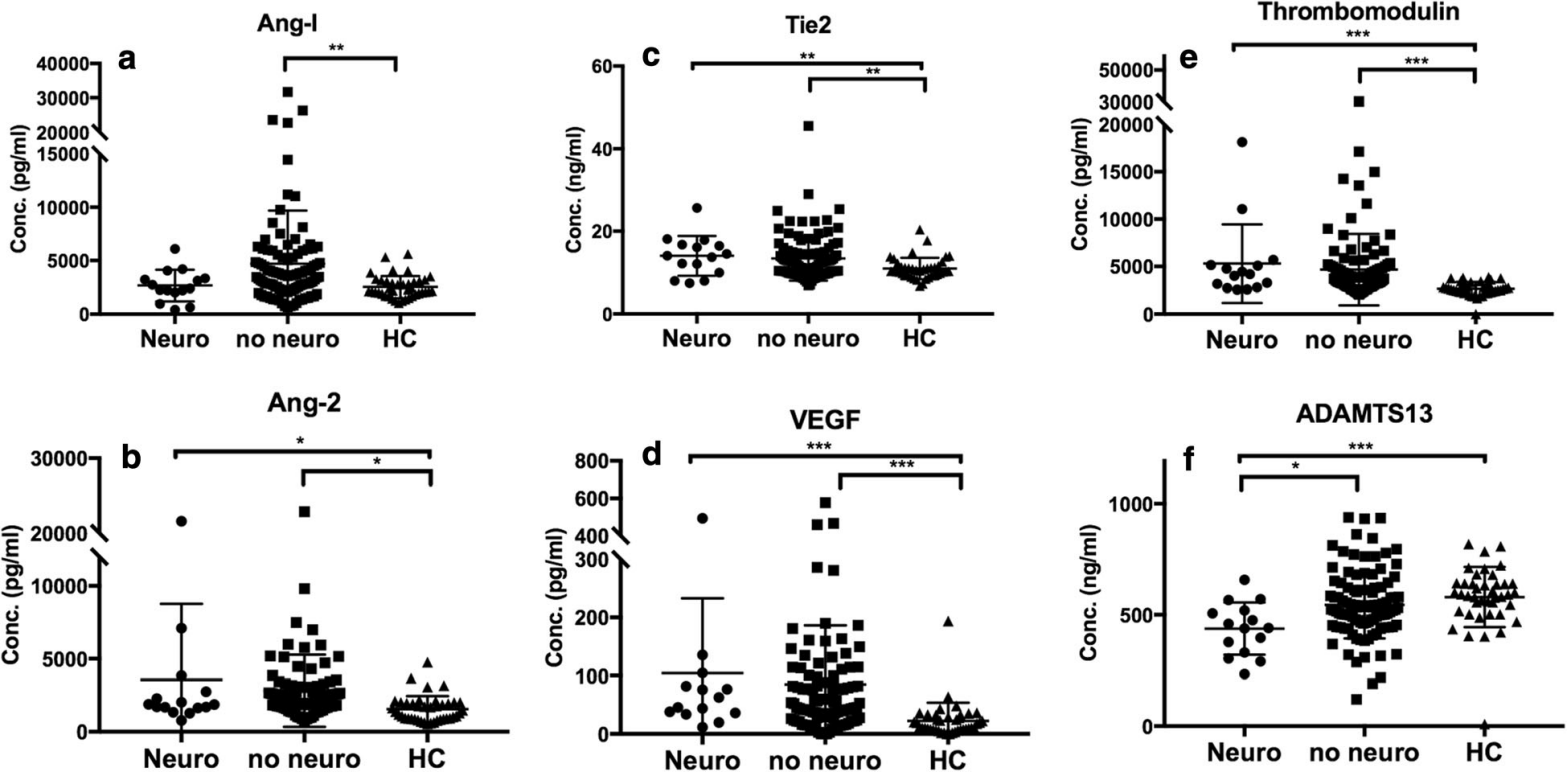
Markers Associated with Neurological Involvement or Not. Serum concentration of ADAMTS13 (F) are significantly lower in pSLE patients with neurological involvement. *p < 0.05, **p < 0.01, ***p < 0.001. **a** Angiopoietin-1; **b** Angiopoietin-2; **c** Tie2; **d** VEGF; **e** Thrombomodulin; **f** ADAMTS13

Among the patients with neurological involvement, 6/15 (40%) had severe headache, 3/15 (20.0%) had limbs numbness and weakness, 3/15 (20%) had psychosis and hallucination, 1/15 (6.7%) had personality change, 1/15 (6.7%) had hearing loss and vertigo, 1/15 (6.7%) had seizure, and 1/15 (6.7%) had urinary incontinence related to myelopathy. All of these manifestations cannot be explained by other etiology other than lupus. 5/15 (33.3%) of them received cerebral single-photon emission computed tomography (SPECT) exam and showed picture of vasculitis and 1/15(6.7%) received brain magnetic resonance angiography (MRA), which is suggestive of vasculitis. Serum concentrations of ADAMTS13 were significantly lower in pSLE patients with neurological involvement compared to those without (437.8 ng/ml ± 30.39, *n* = 15 vs 544.7 ng/ml ± 14.94, *p* < 0.05) (Fig. 4).

### Biomarkers associated with renal involvement in pSLE

**Fig. 5:**
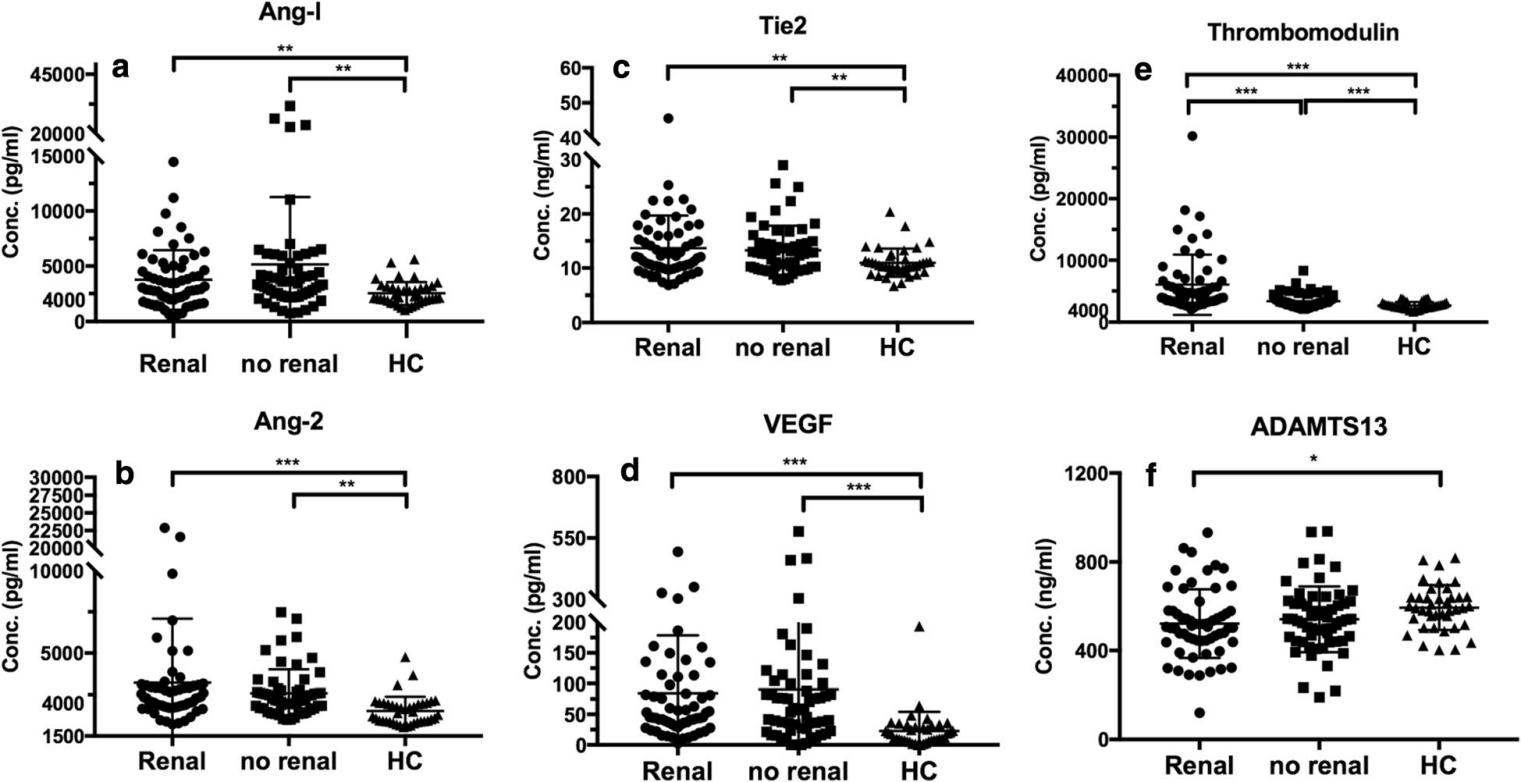
Markers Associated with Renal Involvement or Not Serum thrombomodulin (E) are significantly higher in pSLE patients with renal involvement. *p < 0.05, **p < 0.01, ***p < 0.001. **a** Angiopoietin-1; **b** Angiopoietin-2; **c** Tie2; **d** VEGF; **e** Thrombomodulin; **f** ADAMTS13

Focused on pSLE renal involvement, our data suggested that serum concentrations of thrombomodulin were significantly higher in pSLE patients with renal involvement compared to those without (6055 pg/ml ± 628.8, *n* = 60 vs 3416 ± 148, *n* = 58, *p* < 0.001) (Fig. 5). We also looked into the association of our biomarkers with ISN/RPS (2003) renal histopathological classification and NIH activity index scores, but none of the markers can genuinely reflect lupus nephritis subclasses. (Additional file 2: Table S2, Additional file 3: S4).

**Fig. 6:**
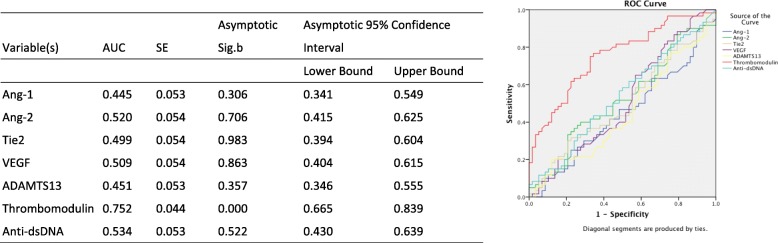
Predictive Value of Biomarkers in Renal involvement Compared to Anti-dsDNA. The ROC curve of these markers compared to Anti-dsDNA in renal involvement in pSLE patients. Light Blue: Thrombomodulin. Dark Blue: Anti-dsDNA

To further elucidate the predictive value of thrombomodulin in pSLE renal involvement, we used the ROC curve to analysis our biomarkers as well as anti-dsDNA, an autoantibody with great correlation with the presence of lupus nephritis [20, 21]. We discovered that thrombomodulin at a level higher than 3333.6 pg/ml, out-performed all biomarkers including anti-dsDNA in predicting renal involvement. (Fig. 6) (AUC = 0.752; *p* < 0.01).

## Discussion

Pediatric-onset SLE was associated with more frequent lupus nephritis whereas nephritis was less common in adult-onset SLE. Besides, there was a trend towards the pediatric-onset SLE cohort having more neurological disease [11]. These data confirm an aggressive phenotype of disease in patients with onset of SLE in childhood and adolescence and supports the need for intensive follow-up and therapy in this population. To search for early treatment and intensive disease monitoring, we aimed to elucidate the association and possible pathogenesis underlining SLE disease activity and organ involvement of endothelial dysregulation. We carefully examined a panel of endothelial dysregulation biomarkers in patients with pSLE. This is the first study analyzing biomarkers associated with endothelial dysregulation in pediatric-onset SLE. In a pediatric SLE cohort of 118 patients, we discovered that serum levels of VEGF, Tie2, thrombomodulin were significantly higher while serum ADAMTS13 was lower in active pSLE patients when compared to those with inactive diseases (all *p* < 0.05). In addition, serum thrombomodulin level was higher in pSLE patient with renal involvement, and serum ADAMTS13 levels was negatively associated with neurological involvement (*p* < 0.05). A cutoff of thrombomodulin at 3333.6 pg/ml and 4346.79 pg/ml best correlated renal involvement and correlated pSLE disease activity respectively.

Ang-1 and Ang-2 are antagonistic ligands which bind to the extracellular domain of the tyrosine kinase and epidermal growth factor-like domains 2 (Tie2) receptors exclusively expressed on endothelial cells. Ang-2 was proposed to be a key mediator of endothelial cell activation that will facilitate endothelial inflammation. Non-redundant constitutively operational Ang-1/Tie2 signaling maintains vessel integrity, inhibits vascular leakage, suppresses inflammatory gene expression and prevents recruitment and transmigration of leukocytes [22]. Considering the known anti-inflammatory effect of Ang-1 on murine vasculature [23], the elevating of Ang-2 and Tie2 but Ang-1 suggested a tilt of balance toward endothelial cell activation and inflammation. Additionally, given a growing consensus on the role of vascular inflammation in promoting atherogenesis and the progression of atherosclerosis [24, 25], chronic Ang-2-driven endothelial activation may play an important role in the increased risk of accelerated cardiovascular diseases (CVD) in those with poor disease control [26, 27].

Vascular endothelial growth factor (VEGF), a member of the angiogenic factor superfamily, also known as vascular permeability factor, is both a potent enhancer of microvascular permeability and a selective endothelial cell growth factor [28]. Although an increased level of this angiogenic molecule has been repeatedly shown among SLE patients with active disease status as compared to those inactive [29, 30], the exact pathologic role of VEGF in SLE still requires further clarification. Our data is in agreement with previous reports on the fine correlation of VEGF with SLE disease activity, however, the association with extensive organ involvements, such as lupus nephritis, antiphospholipid syndrome, vasculitis, and skin manifestation were not seen [28, 29]. Considering the consistency of VEGF in SLE disease status and the possible role of VEGF in extensive organ involvement among cases with SLE, perhaps the use of anti-VEGF would provide further answer to its pathological role and serve as a potential solution for patients with SLE.

Thrombomodulin (TM) is a thrombin receptor expressed on the vascular endothelial cell surface. It inhibits the procoagulant activities of thrombin and is a cofactor for the thrombin-catalyzed activation of protein C. As demonstrated in vitro, serum TM is most likely released upon endothelial cell damage [31]. The impairment in either the quantity or quality of TM could possibly play a pathogenic role in thrombogenesis which is commonly observed in patients with SLE [32]. Among all biomarkers, including the well-characterized serological parameters anti-dsDNA, thrombomodulin shows the best correlation to disease activity. Proposed as a marker for endothelial cell damage, thrombomodulin has been associated with active vasculitis in SLE and correlated significantly and positively with SLEDAI score [33].

ADAMTS13 (a disintegrin and metalloproteinase with a thrombospondin type 1 motif, member 13) is a protease synthesized by human stellate hepatic cells and vascular endothelial cells, which cleaves specifically ultra-large von Willebrand Factor (VWF) multimers at the VWF-A2 domain, generating shorter globular multimers in the normal circulation. A lack of proteolytic activity results in the presence of unusal hyper-adhesive large VWF strings on the endothelial cell surface, which are more likely to bind to platelets than normal globular VWF, leading to platelet aggregation and thrombus formation in the microvasculature [34, 35]. Previous study show that SLE is a pathological condition with mild to moderate ADAMTS13 activity deficiency and high levels of VWF and vascular cell adhesion molecule 1 (VCAM-1), potentially related to endothelial damage [36]. ADAMTS13 activity in SLE is specially reduced in patients with active disease and in those with antiphospholipid antibodies [36]. There was, however, no association seen between reduced ADAMTS 13 serum concentrations with antiphospholipid antibodies in our cohort.

Unlike previous study [37], our data suggested that the levels of ADAMTS13 were significantly lower in pSLE patients than in normal controls and negatively correlate SLEDAI. Although VWF was not directly measured in the present study, studies have reported increased VWF protein levels in patients with SLE [38]. The controversy between our data with previous observation remains speculative at the moment. Considering its pathologic role and its associating with VWF, however, our data strongly argue that ADAMTS13, to be a contradictory marker for SLE disease activity.

Lupus nephritis (LN) is a severe manifestation that is commonly recognized in patients with SLE [39, 40]. Classically, LN has been considered an immune complex-induced microvascular injury, initiated by renal deposit of circulating anti-dsDNA complexes, in situ cross-reactive antibodies and the secondary recruitment of various immune cells [41]. Ang-2 [42], VEGF and thrombomodulin have all been described to associate with renal involvement [43–47]. Without much surprise, none of the markers were reported to correlate the histopathologial classification categorized by the ISN/RPS systems, which focus thoroughly on renal glomerulopathy [48, 49]. In the present study, the elevation of thrombomodulin in cases with kidney involvement may be explained by a possible pro-thrombotic tendency and sequential endothelium injury. Furthermore, knowing that thrombomodulin is excreted via kidneys perhaps explanation why it stool out from all markers.

The etiology of neurophysiatric manifestations in SLE was considered to be multifactorial, possibly involving autoantibody production, microangiopathy, intrathecal production of proinflammatory cytokines and premature atherosclerosis [50, 51]. ADAMTS13 was significantly lower in pSLE patients with neurological involvement compared to those without. Although thrombotic event is likely to contribute the pathogenesis, no histological or radiologic evidence was observed in our present cohort.

The strength of our study lies in the comprehensive and simultaneous analysis of clinical and serological data from a well-defined cohort study. Nonetheless, there are several limitations in the present study. First, this study was not aimed at elucidating specific pathophysiological pathways of biomarkers regulation in SLE, but was rather designed to evaluate a possible clinical significance and features compared to these biomarkers. Second, by choosing a cross-sectional design, we cannot rule out excess circulating biomarkers as a consequence rather than a cause of these presentations.

Our study have shown that endothelial dysregulation associating biomarkers can serve as potent biomarkers associating pSLE activity as well as renal and neurophysiatric involvement. Further investigation and clarification of these biomarkers will provide a better understanding of the vascular pathogenesis in SLE.

## Conclusions

Endothelial dysregulation associating proteins seems to be potent biomarkers for pSLE activity as well as organ involvement in pSLE patients. These biomarkers may be beneficial in understanding of the vascular pathogenesis and disease monitoring.

## Supplementary information

**Additional file 1: Figure S1.** Markers Associated with Disease Activity (active v.s. inactive) using Cutoff Level of SLEDAI 7. *p<0.05, **p<0.01, ***p<0.001, ****p<0.0001. **Figure S2.** Predictive Value of Biomarkers in Renal. Involvement Compared to Complement level. Abbreviations: AUC, area under the curve; SE, standard error.

**Additional file 2: Table S1.** Markers Associated with C3 and C4. **Table S2.** Markers associated with NIH Activity Index. **Table S3.** Markers in Four SLEDAI Subgroups.

**Additional file 3: Table S4.** Markers among the WHO Classifications of Lupus Nephritis.

## Data Availability

The datasets used and/or analyzed during the current study are available from the corresponding author on reasonable request.

## Abbreviations

Activity Index; CNS: Central Nervous System
ADAMTS13: A Disintegrin-like and Metalloprotease with Thrombospondin Type 1 Motif
Ang-1: Angiopoietin-1
Ang-2: Angiopoietin-2;
anti-dsDNA: anti-double stranded DNA
AUC: Area under the curve
CVD: Cardiovascular diseases
Factor; pSLE: Pediatric-onset Systemic Lupus Erythematosus
SLEDAI: SLE Disease
LN: Lupus Nephritis
TM: Thrombomodulin; EC: Endothelial Cell
TMA: Thrombotic Microangiopathy;
VCAM-1: Vascular Cell Adhesion Molecule 1
VEGF: Vascular Endothelial Growth
VWF: Von Willebrand Factor
