# Correction to: Biomarkers associating endothelial dysregulation in pediatric-onset systemic lupus erythematous

**DOI:** 10.1186/s12969-020-0405-7

**Published:** 2020-02-18

**Authors:** Wan-Fang Lee, Chao-Yi Wu, Huang-Yu Yang, Wen-I Lee, Li-Chen Chen, Liang-Shiou Ou, Jing-Long Huang

**Affiliations:** 1 Division of Allergy, Asthma, and Rheumatology, Department of Pediatrics, Chang Gung Memorial Hospital Linko branch, Taoyuan, Taiwan; 2 Chang Gung University, College of Medicine, Taoyuan, Taiwan; 3 Department of Nephrology, Chang Gung Memorial Hospital Linko branch, Taoyuan, Taiwan

**Correction to: Pediatr Rheumatol Online J (2019) 17:69**

**https://doi.org/10.1186/s12969-019-0369-7**

Following publication of the original article [1], we have been notified that the colour representation of the graph is not correct in Figure 6 legend.

The legend of Figure 6 should state the below:

Fig. 6 Predictive Value of Biomarkers in Renal involvement Compared to Anti-dsDNA. The ROC curve of these markers compared to Anti-dsDNA in renal involvement in pSLE patients. Red: Thrombomodulin. Light Blue: Anti-dsDNA
