# Supplementary material for: Biomarkers associating endothelial Dysregulation in pediatric-onset systemic lupus erythematous
Source: Pediatr Rheumatol Online J. 2019 Oct 24;17:69. doi: 10.1186/s12969-019-0369-7 (PMC6814049; doi:10.1186/s12969-019-0369-7)
Supplement: Supplementary file 1 — Additional file 1: Figure S1. Markers Associated with Disease Activity (active v.s. inactive) using Cutoff Level of SLEDAI 7. *p<0.05, **p<0.01, ***p<0.001, ****p<0.0001. Figure S2. Predictive Value of Biomarkers in Renal. Involvement Compared to Complement level. Abbreviations: AUC, area under the curve; SE, standard error. [file 12969_2019_369_MOESM1_ESM.docx]

**
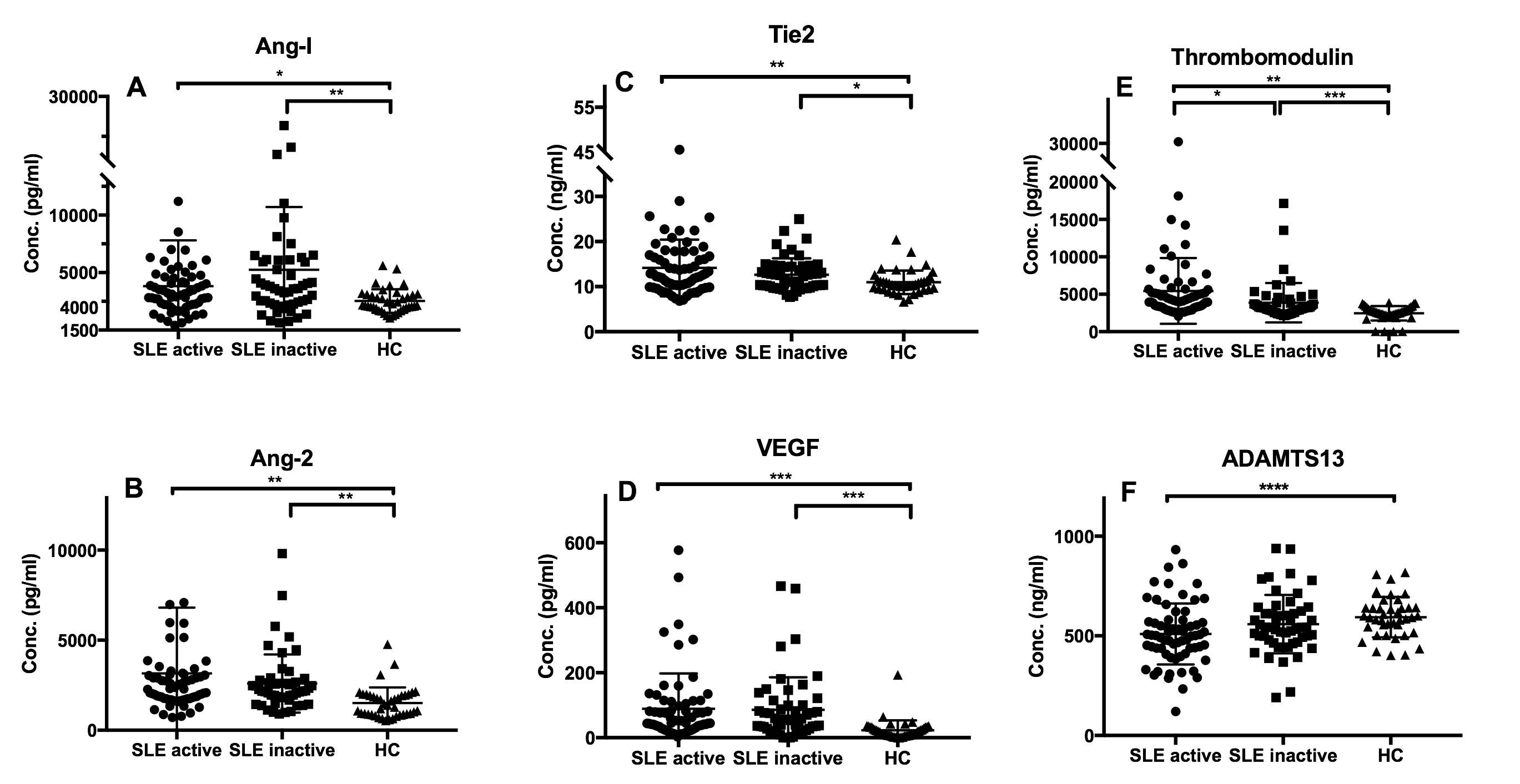
Figure S1. Markers Associated with Disease Activity (active v.s. inactive) using Cutoff Level of SLEDAI 7**

****p*<0.05, ***p*<0.01, ****p*<0.001, *****p*<0.0001**


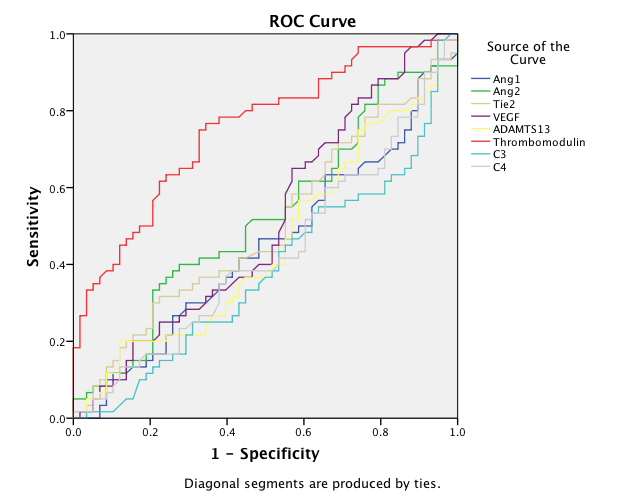


| Variable(s) | AUC | SE | Asymptotic Sig.b | Asymptotic 95% Confidence Interval | |
| --- | --- | --- | --- | --- | --- |
|  |  |  |  | Lower Bound | Upper Bound |
| Ang-1 | 0.445 | 0.053 | 0.306 | 0.341 | 0.549 |
| Ang-2 | 0.520 | 0.054 | 0.706 | 0.415 | 0.625 |
| Tie2 | 0.499 | 0.054 | 0.983 | 0.394 | 0.604 |
| VEGF | 0.509 | 0.054 | 0.863 | 0.404 | 0.615 |
| ADAMTS13 | 0.451 | 0.053 | 0.357 | 0.346 | 0.555 |
| Thrombomodulin | 0.752 | 0.044 | 0.000 | 0.665 | 0.839 |
| C3 | 0.385 | 0.052 | 0.032 | 0.284 | 0.487 |
| C4 | 0.429 | 0.053 | 0.181 | 0.325 | 0.532 |

**Figure S2. Predictive Value of Biomarkers in Renal**

**Involvement Compared to Complement level**

**Abbreviations: AUC, area under the curve; SE, standard error**
