# Supplementary material for: Biomarkers associating endothelial Dysregulation in pediatric-onset systemic lupus erythematous
Source: Pediatr Rheumatol Online J. 2019 Oct 24;17:69. doi: 10.1186/s12969-019-0369-7 (PMC6814049; doi:10.1186/s12969-019-0369-7)
Supplement: Supplementary file 2 — Additional file 2: Table S1. Markers Associated with C3 and C4. Table S2. Markers associated with NIH Activity Index. Table S3. Markers in Four SLEDAI Subgroups. [file 12969_2019_369_MOESM2_ESM.docx]

**Table S1. Markers Associated with C3 and C4**

**p*< 0.05


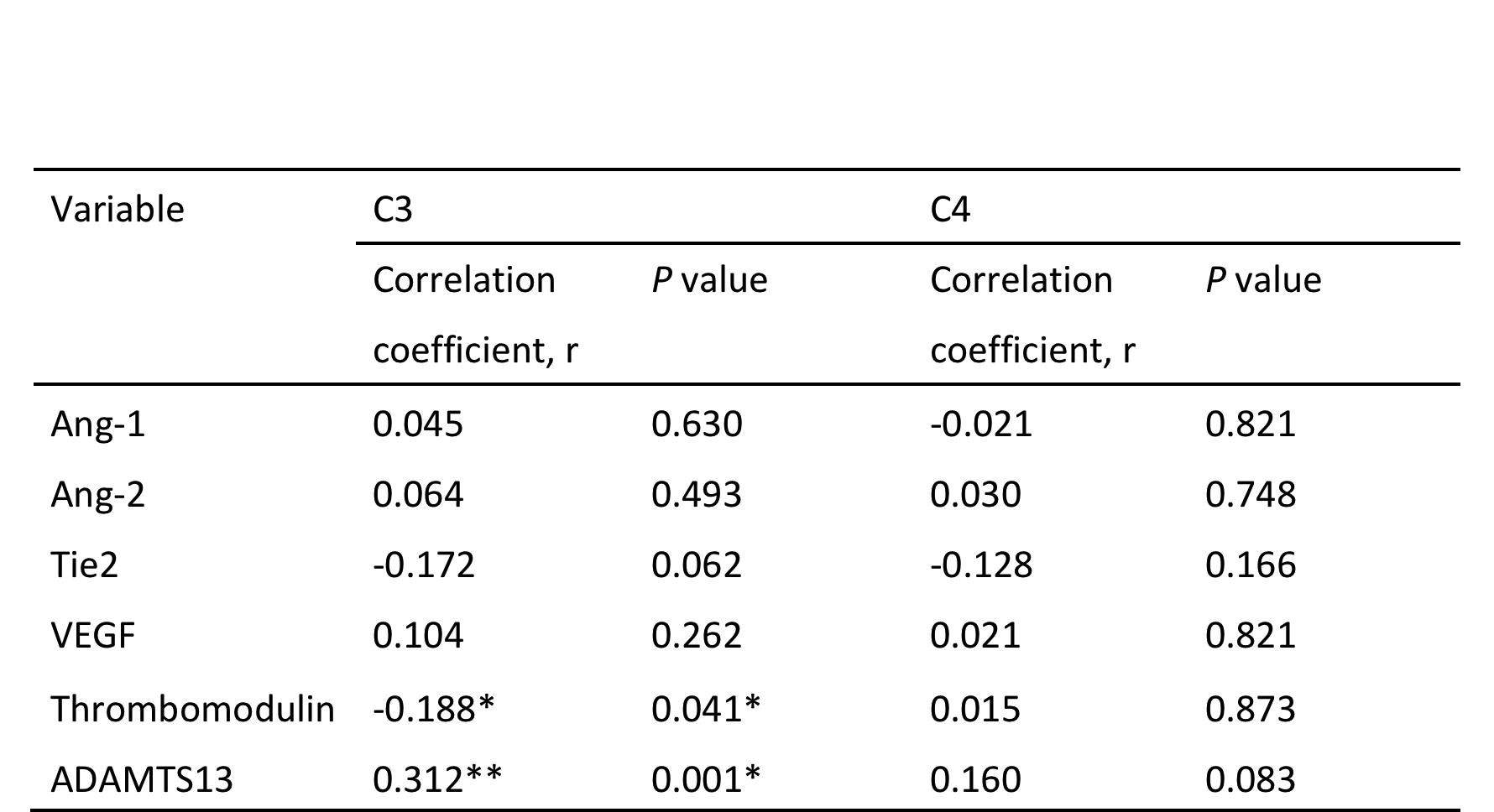


**Table S2. Markers associated with NIH Activity Index**


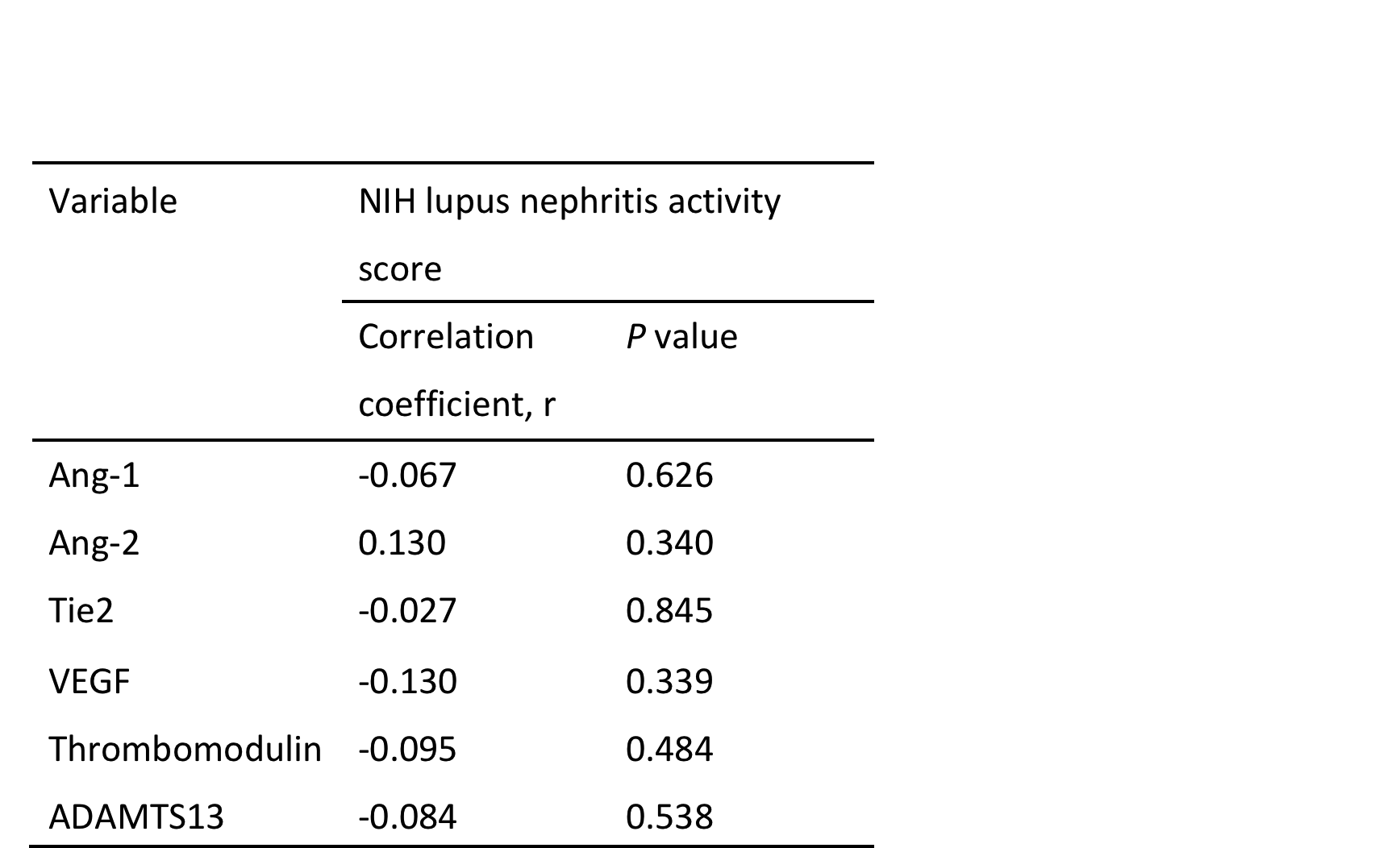


**Table S3. Markers in Four SLEDAI Subgroups**

**Group 1: 0≤ SLEDAI≤4**

**Group 2: 4< SLEDAI≤7**

**Group 3: 7< SLEDAI≤12**

**Group 4: 12<SLEDAI**
