# Supplementary material for: Biomarkers associating endothelial Dysregulation in pediatric-onset systemic lupus erythematous
Source: Pediatr Rheumatol Online J. 2019 Oct 24;17:69. doi: 10.1186/s12969-019-0369-7 (PMC6814049; doi:10.1186/s12969-019-0369-7)
Supplement: Supplementary file 3 — Additional file 3: Table S4. Markers among the WHO Classifications of Lupus Nephritis. [file 12969_2019_369_MOESM3_ESM.docx]

| Variable  mean±SD | II  (n=8) | III  (n=12) | IV  (n=44) | V  (n=4) | III+V  (n=3) | IV+V  (n=2) | *P* value |
| --- | --- | --- | --- | --- | --- | --- | --- |
| Ang-1 (pg/ml) | 3717.19±1690.61 | 3057.10±1485.28 | 3965.29±2996.90 | 3606.98±3228.74 | 6283.55±2360.50 | 3040.97±418.53 | 0.627 |
| Ang-2 (pg/ml) | 3557.25±1823.14 | 1978.27±627.02 | 2874.55±3337.38 | 4042.09±3969.61 | 1953.57±450.51 | 5102.18±2807.53 | 0.736 |
| Tie2 (ng/ml) | 16.69±11.99 | 13.21±4.05 | 12.78±4.17 | 10.79±2.85 | 10.70±2.03 | 13.21±6.56 | 0.410 |
| VEGF (pg/ml) | 77.22±52.66 | 86.65±63.04 | 73.44±70.32 | 38.35±25.37 | 192.91±240.42 | 34.67±5.15 | 0.596 |
| ADAMTS13  (ng/ml) | 567.94±186.68 | 549.79±140.11 | 507.14±137.89 | 602.69±83.56 | 508.15±188.61 | 486.85±65.67 | 0.815 |
| Thrombomodulin  (pg/ml) | 4559.24±3007.49 | 4359.05±1593.51 | 6154.75±5422.32 | 3617.83±547.39 | 7353.64±6679.94 | 3571.27±1104.34 | 0.225 |

**Table S4. Markers among the WHO Classifications of Lupus Nephritis**
